# Workplace family support perception, work-family balance, job and family satisfaction among employed mothers and fathers in Chile

**DOI:** 10.3389/fpsyg.2025.1554094

**Published:** 2025-05-20

**Authors:** Leonor Riquelme-Segura, Berta Schnettler, Marisa Matias, Andrés Concha-Salgado, Ligia Orellana, José A. Sepulveda

**Affiliations:** ^1^Departamento de Trabajo Social, Facultad de Educación, Ciencias Sociales y Humanidades, Universidad de La Frontera, Temuco, Chile; ^2^Centro de Excelencia en Psicología Económica y del Consumo (CEPEC), Universidad de La Frontera, Temuco, Chile; ^3^Facultad de Ciencias Agropecuarias y Medioambiente, Universidad de La Frontera, Temuco, Chile; ^4^Scientific and Technological Bioresource Nucleus (BIOREN-UFRO), Universidad de La Frontera, Temuco, Chile; ^5^Universidad Católica de Santiago de Guayaquil, Guayaquil, Ecuador; ^6^Center for Psychology and Faculty of Psychology and Educational Sciences, University of Porto, Porto, Portugal; ^7^Departamento de Psicología, Facultad de Educación, Ciencias Sociales y Humanidades, Universidad de La Frontera, Temuco, Chile

**Keywords:** workplace family support, work-family balance, work and family wellbeing, employed, mothers, fathers

## Abstract

**Introduction:**

Dual-income families with children face the challenge of balancing work and family to achieve greater wellbeing, especially in the context of the health emergency resulting from the COVID-19 pandemic. A quantitative methodology was used to examine the direct and indirect intraindividual and interindividual relationships between the workplace family support perception, work-family balance, and job and family satisfaction.

**Methods:**

The study involved 454 couples of mothers and fathers of children between 12 and 16 years of age, workers from two regions in Chile. The questionnaires included the scales of perceived workplace support for families, work-family balance, job satisfaction, and family satisfaction.

**Results:**

The results provide new insights into the positive direct and indirect link between workplace family support perceptions, work-family balance, and satisfaction with work and family domains in mothers and fathers with paid work. In addition, they show the role of work-family balance as an underlying mechanism through which greater job satisfaction (at the intra- and interindividual level) and family satisfaction (at the intra- and interindividual level) would be possible.

**Discussion:**

New insights are provided on the direct and indirect positive relationship with symmetrical crossovers in mothers and fathers between perceived workplace support for the family, work-family balance, job satisfaction, and family satisfaction.

## 1 Introduction

Balancing work and family life is becoming a major concern for today’s workforce (Hirschi et al., 2019). Families are increasingly confronted with difficulties such as time constraints and obstacles to balancing work and family life, which constitute a persistent source of stress for families with children (Bernardi et al., 2017). While this occurs in families at different stages of the family life cycle, it represents different dynamics in families with adolescent children. Adolescents pursue the search for autonomy and independence, challenge the authority of their parental figures (Alonso and Aliaga, 2017), have distinct needs, and require different forms of support than children in previous stages (Matias and Recharte, 2020). In addition, these stage of the life cycle also poses new challenges for parental wellbeing (Meier et al., 2018).

Throughout the COVID-19 pandemic, couples struggled to reconcile work and family responsibilities due to changes in the employment landscape (Kramer and Kramer, 2020) and the reinforcement of conventional gender norms (Vitória et al., 2022). New demands of job and family coexisted in the same physical space (Barriga Medina et al., 2021) as a result of the sudden installation of remote work, without ensuring access to technology, connectivity, or giving workers the necessary training (Vasic, 2020), alongside care responsibilities due to school closures (Macartney et al., 2020). This situation was unprecedented and putted families under high pressure to balance work and family roles and maintain subjective wellbeing. Studying this process in an emergency or contingency situation is relevant and may inform future scenarios.

A person’s subjective wellbeing comprises a cognitive component called life satisfaction, and an affective component called happiness, which refers to positive feelings (Diener, 2000). For Diener et al. (1999), life satisfaction refers to a person’s positive assessment of their life in general or of particular aspects, such as work, family, or health. Accordingly, as people would be transiting between paid work and family, it becomes relevant to delve into job satisfaction which implies how much an individual likes his or her job (Agho et al., 1992), as well as family satisfaction which refers to a person’s conscious cognitive judgment of his or her family life (Zabriskie and McCormick, 2003).

The literature has identified Hobfoll (1989) Conservation of Resources Theory (COR) as one of the most encompassing approaches to understanding how enhanced resources can facilitate a more favorable work-family balance (Hobfoll et al., 2018). This theory postulates that resources, which could be objects, conditions, personal characteristics, or even energy (Hobfoll and Shirom, 2000), a person obtains would help them cope with contextual demands and could also lead them to generate more resources through the existence of a gain spiral process where resources accumulate (Hobfoll, 1989).

Hobfoll (1989) states that the influence of the resources gained will be sustained over time and in different circumstances, so that the resources a person can obtain from their work will lead to their acquisition, which can be used to improve performance in other domains of life. Therefore, as the resources an individual generates in the workplace enable them to function more effectively, there is an investment, which translates into a commitment to activities that require resources, such as problem-solving. This would contribute to better work or family functioning, which in turn would generate more resources. In this sense, a person who works for pay can utilize, for example, flexible hours to perform their work more efficiently, saving time that can be allocated to other domains, such as work or family.

A relevant dimension of COR theory is resource sharing based on the *Crossover* Model of Chen et al. (2015), which involves the interindividual transmission of resources between members of a dyad or couple (Hobfoll et al., 2018). *Crossover* involves the interindividual transmission of demands, stress, or positive experiences between domains, crossing people closest to each other and share the same environment (Westman, 2001). For Kenny et al. (2006), such transmission is referred to as the companion effect. According to Westman (2001), positive feelings about work events could be transmitted to the partner and positively affect their wellbeing (Bakker et al., 2009).

In mediation research, two effects can be distinguished within the resource transfer paradigm. On the one hand, the *cross-domain* effect refers to one effect influencing or crossing another domain (Frone et al., 1992); for example, resources from work are transferred to the family, which results in greater family satisfaction. On the other hand, there is the *matching* effect *within* the domain, which refers to the main effect being in the domain where the resource is generated (Amstad et al., 2011); for example, resources from work are transferred to the family, but the individual reacts with positive emotions toward the domain in which these resources are generated, thereby enhancing job satisfaction in this scenario.

A work resource identified in the literature is support for the family at work (Matias et al., 2017; Yucel and Minnotte, 2017). Matias et al. (2017) describe the flexibility in work schedules, prioritizing vacations, and allowing parents to attend medical appointments and school meetings as practices in workplace family support. It originates from the organization and is the most important source of support, surpassing supervisor support and coworker support (French et al., 2018). This form of work support has demonstrated a beneficial effect by alleviating psychological stress and fostering balance between the professional and family domains (Drummond et al., 2017; Pattusamy and Jacob, 2017; Yucel and Minnotte, 2017).

For Grzywacz and Carlson (2007), the balance between work and family domains entails fulfilling role-specific expectations that are negotiated and mutually agreed upon by an individual and their role partners within each domain. This concept of work-family balance is rooted in Marks and MacDermid’s (1996) Theory of Role Balance. According to this theory, balance does not rely on prioritizing certain roles or on performance within those roles. Instead, it emerges from ongoing and flexible negotiations related to those roles, highlighting the inherently social nature of people’s responsibilities (Grzywacz and Carlson, 2007). Individuals who achieve a better balance between different roles, such as work and family, experience higher levels of wellbeing (Marks and MacDermid, 1996). This is because they are more able to engage fully in each role, recognizing that all roles are equally important.

However, most of the studies on the work-family interface assessing work-family balance conducted during the pandemic were conducted in the European Union, North America, and Asia (Vitória et al., 2022) and at the individual level, without considering the interdependence between the members of a couple as proposed by Kerr and Bowen’s Family Systems Theory (Kerr and Bowen, 1988). This theory of human behavior aims to describe the relationships that form within families. It acknowledges the interdependence among individuals, who, through reciprocal relationships, become emotionally connected and can influence one another’s thoughts, emotions, and behaviors. This dynamic is commonly observed in family settings (Kerr and Bowen, 1988). In addition, most research examining couples has done so with samples of preschool-aged children (Matias et al., 2017) and has not considered matching effect mediation approaches (Amstad et al., 2011) or the impact across different domains (Frone et al., 1992).

This study aims to enhance the existing literature, particularly within the Latin American context, by examining how perceptions of workplace family support and work-family balance can influence satisfaction in both job and family domains. Additionally, we will conduct a thorough investigation of work-family balance as a mediating variable that may contribute to the wellbeing of couples. Therefore, in this study, we examined the direct and indirect intraindividual (actor effect) and interindividual (partner effect) relationships among workplace family support perception, work-family balance, and job and family satisfaction in employed mothers and fathers with adolescent children.

### 1.1 Direct associations between workplace family support perception, work-family balance, and job and family satisfaction

Family-friendly organizational policies enable employees to manage work and family responsibilities (Schulz-Knappe and Ter Hoeven, 2020). In turn, supportive work environments would benefit dual-earner families (Lawson et al., 2019), allowing them to better balance their multiple roles (Gahlawat et al., 2019). Using a qualitative methodology, Syed et al. (2022) found that supportive practices, such as flexible working hours, are more important than providing financial benefits to balance work and family.

Studies report that organizational support is positively and significantly associated with the work-life balance of paid workers in banking companies (Fitria and Linda, 2019) and that the greater the organizational support, the greater the work-family balance in Italian couples (Lo Presti et al., 2020, 2022). Similarly, the perception of workplace family support affects the parental satisfaction of paid workers (Matias et al., 2017) and organizational support increases job satisfaction (Kurtessis et al., 2017; Mascarenhas et al., 2022), effectiveness, and family functioning (Gahlawat et al., 2019; Mesimo-Ogunsanya, 2017; Tang et al., 2014).

Despite evidence relating work resources to a better work-family balance and both work and family satisfaction, there is limited evidence about the transfer of work resources between couple members, i.e., the transference of a resource originating from one partner’s work affecting the other partner work-family balance or family satisfaction. In this regard, Brady et al. (2021) suggest that resources derived from workplace family support perceptions are transferred between partners. Similarly, with a sample of Chilean couples with dual parental income, Schnettler et al. (2022) found that resources acquired by the father in the workplace were transferred to the mother, improving her diet quality. One might then expect that a partner’s perception of family support in the workplace can act as a resource that enhances their own work-family balance, job satisfaction, or family satisfaction and also have a positive link to the other partner’s work-family balance, job satisfaction, or family satisfaction.

Given the above, the following hypotheses are posited:

*Hypothesis 1 (H1):* Workplace family support perception is positively associated with work-family balance in both partners (actor effect).

*Hypothesis 2 (H2):* Workplace family support perception of one member of the couple is positively associated with the partner’s work-family balance (partner effect).

*Hypothesis 3 (H3):* Workplace family support perception is positively associated with job satisfaction in both partners (actor effect).

*Hypothesis 4 (H4):* Workplace family support perception of one member of the couple is positively associated with the partner’s job satisfaction (partner effect).

*Hypothesis 5 (H5):* Workplace family support perception is positively associated with family satisfaction in both partners (actor effect).

*Hypothesis 6 (H6):* Workplace family support perception of one member of the couple is positively associated with the partner’s family satisfaction (partner effect).

Compared to studies on work–family conflict and work-family enrichment, fewer studies have focused on work-family balance (Landolfi and Lo Presti, 2020). However, research on work-family balance has identified a correlation with wellbeing across various life domains for both men and women as both genders navigate the demands of multiple roles. Thus, it has been reported that work-family balance is associated with greater job satisfaction in different countries (Carlson et al., 2009; Ferguson et al., 2012; Zhang et al., 2020; Leung et al., 2020; Raza et al., 2018) including Chile (Chiang et al., 2022, Chiang Vega et al., 2024). Studies also relate work-family balance to greater family satisfaction in workers from different countries (Carlson et al., 2009; Ferguson et al., 2012; Leung et al., 2020) as well as in Chilean workers (Riquelme-Segura et al., 2023).

The crossover effect involves the interindividual transmission of resources between members of a dyad (Hobfoll et al., 2018) and explains how experiences are transferred between closely related individuals (Westman, 2001). Crossovers could be bidirectional or symmetrical and one-dimensional or asymmetrical (Mauno et al., 2017; Orellana et al., 2021). Primarily, in cultures where traditional roles are more evenly distributed, the crossover effect occurs in a unidirectional manner, from men to women. Meanwhile, in cultures where the ideology regarding gender role is more egalitarian, the crossover effect would be symmetrical and bidirectional, from man to woman and from woman to man (Westman, 2005). Therefore, one could expect the existence of different patterns in crossovers associated with gender, depending on the country’s culture.

Studies focused on the transfer of resources derived from the work-family balance are limited. To the best of the authors’ knowledge, there are no available studies in Chile. However, with a sample of dual-earner couples in the United States, Wan et al. (2021) identified crossover effects in which one partner’s work-family balance improves the other’s work-family balance. In Chile, traditional roles still prevail. Therefore, we expect that the work-family balance experienced by one member of the couple can lead to job satisfaction and family satisfaction in the other, at least in a unidirectional way.

Therefore, we propose the following hypotheses:

*Hypothesis 7 (H7):* Work-family balance is positively associated with job satisfaction in both partners (actor effect).

*Hypothesis 8 (H8):* One partner’s work-family balance is positively associated with the other partner’s job satisfaction (partner effect).

*Hypothesis 9 (H9):* Work-family balance is positively associated with family satisfaction in both partners (actor effect).

*Hypothesis 10 (H10):* One partner’s work-family balance is positively associated with the other’s family satisfaction (partner effect).

### 1.2 Mediating role of work-family balance

Mediation occurs when one variable influences another variable via an intermediary variable, such as work-family balance, resulting in an indirect effect (Hayes, 2018). Specifically, this study refers to the increased workplace family support perception, which affects work-family balance first and then job and family satisfaction.

The role of work-family balance as a mediator has been previously examined in the literature, although with less research development (Landolfi et al., 2021; Raza et al., 2018), demonstrating its mediation between social support and job and family satisfaction in a study with American workers (Ferguson et al., 2012). Likewise, its mediating role between family support for work issues and job satisfaction was determined in Asian workers (Zhang et al., 2020). In French self-employed workers, work-family balance was a mediatior between emotional support and job and family satisfaction, as well as between instrumental family support and job and family satisfaction (Leung et al., 2020).

For their part, Raza et al. (2018) reported that in healthcare workers residing in Asia, there is a complete mediation of work-family balance between the trait of mindfulness or being aware of lived experiences in the moment and job satisfaction. Lo Presti et al. (2020) also provide evidence in their study of Italian dual-earner couples, illustrating the mediating function of work-family balance between work-family organizational support and family satisfaction. Qiu and Dauth (2022) showed that work-family balance mediates the relationship between virtual work intensity and job satisfaction in German and Chinese employees performing virtual work. Bobbio et al. (2022) demonstrated that work-family balance mediates the relationship between supervisor support and life satisfaction and between organizational support and positive affective experiences in Italian public and private employees.

During the COVID-19 pandemic, a longitudinal study by Landolfi et al. (2021) reported that work-family balance mediates the relationship between work-family resources (controlling the work, supervisor support, and family support) and life satisfaction in Italian education workers. Similarly, during the pandemic, Pan et al. (2022) showed that work-family balance mediates the relationship between the trait of mindfulness and subjective wellbeing.

Therefore, we expect the work-family balance to serve as a link between workers’ labor resources and their job and family satisfaction, as well as their partners’ job and family satisfaction.

In reference to the above, the mediating role of the work-family balance is explored through the following hypotheses:

*Hypothesis 11 (H11):* Work-family balance mediates between workplace family support perception and job satisfaction in both partners (actor and partner effects).

*Hypothesis 12 (H12):* Work-family balance mediates the relationship between workplace family support perception and family satisfaction in both partners (actor and partner effects).

**Figure 1:**
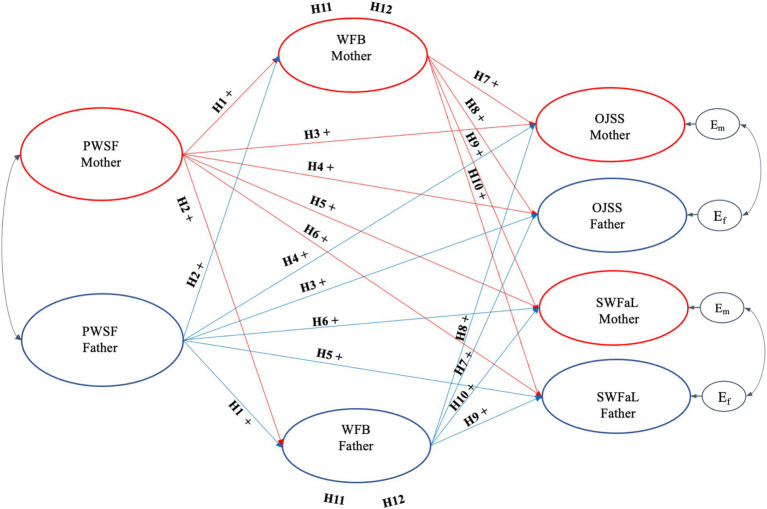
Path diagram of the dyadic structural theoretical model. E_m_: residual errors of job satisfaction and mothers’ family satisfaction. E_f_: residual errors of job satisfaction and fathers’ family satisfaction.

Figure 1 shows the theoretical model evaluated in the study.

Studies that have addressed mediating variables other than work-family balance have identified a cross-domain effect or an effect that influences or crosses into another domain (Drummond et al., 2017; Frone et al., 1992; Schnettler et al., 2022) and a matching effect or main effect that is in the domain where the resource is generated (Amstad et al., 2011; Drummond et al., 2017; Schnettler et al., 2024). Additionally, individual and interindividual mediating functions have been reported where an effect crosses from one domain to another (Carlson et al., 2019; Schnettler et al., 2022), i.e., resources generated in the work domain are transferred to family satisfaction through the work-family balance. At the same time, there is evidence of an indirect effect within the same domain at the individual (Ollier-Malaterre et al., 2020) and interindividual levels (Schnettler et al., 2024), i.e., resources generated in the work domain generate a main effect on job satisfaction through the work-family balance both in the individual and between members of a dyad. On this basis, we expect to detect both cross-domain and matching effects in the proposed indirect relationships.

## 2 Method

The study had a non-experimental and cross-sectional design with a descriptive-correlational-explanatory scope (Ato et al., 2013). It was approved by the Ethics Committee of the Universidad de La Frontera (protocol 007/19). It is part of a larger study that examines work-family-food interrelationships and life satisfaction in Chilean families.

### 2.1 Sample and procedure

The study used a non-probabilistic sample of 454 dyads of fathers and mothers (married or cohabiting). The dyads consisted of mothers and fathers in paid employment, with at least one adolescent child between the ages of 10 and 16, residing in the Metropolitan Region and the La Araucanía Region, Chile. Table 1 depicts the sociodemographic characteristics of the sample. The average ages for mothers and fathers were 39.7 years and 42.1 years, respectively. Most of the dyads belonged to the middle socioeconomic level (84.5%), were married (53.3%), and did not have a university degree (60.2%). Families consisted of an average of four members, including two children. Most of the families belonged to the middle SES. Most mothers and fathers worked full-time (a 45-h workweek is typical in Chile) and worked in person during the data collection period.

**Table 1:** Characteristics of the sample of dual-earner parents (*n* = 454).

Characteristics	La Araucanía Region (*n* = 222)	Metropolitana Region (*n* = 232)	Total sample (*n* = 454)	*p-value*
Number of family members [Mean (SD)]	4.1 (0.8)	4.4 (1.0)	4.2 (0.9)	<0.001
Number of children [Mean (SD)]	2.0 (0.7)	2.1 (0.8)	2.1 (0.8)	0.011
Socioeconomic status (%)				
High	0	12.1	6.2	
Middle	86.0	83.2	84.5	< 0.001
Low	14.0	4.7	9.3	
Workplace family support perception [Mean (SD)]				
Mother	7.7 (2.6)	8.0 (2.7)	7.9 (2.7)	0.376
Father	7.0 (2.8)	7.5 (2.7)	7.3 (2.8)	0.839
Work-family balance [Mean (SD)]				
Mother	24.3 (3.4)	24.5 (3.4)	24.4 (3.4)	0.809
Father	24.7 (3.6)	25.0 (3.4)	24.8 (3.5)	0.351
Job satisfaction [Mean (SD)]				
Mother	21.4 (5.0)	21.2 (5.0)	21.3 (5.0)	0.637
Father	21.3 (5.0)	21.4 (5.1)	21.3 (5.0)	0.710
Satisfaction with family life [Mean (SD)]				
Mother	23.2 (5.1)	24.1 (5.0)	23.7 (5.1)	0.400
Father	23.7 (5.0)	24.6 (5.2)	24.2 (5.1)	0.963
Mothers’ working hours (%)				
45 h per week	61.7	65.5	63.7	0.096
Less than 45 h per week	38.3	34.5	36.3	
Fathers’ working hours (%)				
45 h per week	82.0	78.4	80.2	0.059
Less than 45 h per week	18.0	21.6	19.8	
Mothers’ work modality (%)				
On-site at work	80.6	44.8	62.3	<0.001
Telework at home	19.4	55.2	37.7	
Fathers’ work modality (%)				
On-site at work	91.9	66.4	78.9	<0.001
Telework at home	8.1	33.6	21.1	

It is worth noting that Chile is composed of 16 regions, which exhibit significant social and economic differences, as exemplified by the Metropolitan Region and the Region of La Araucanía. For further background, the Metropolitan region has a poverty rate of 4.4% (MDS, Ministry of Social Development, 2023) and also boasts the highest Human Development Index in the country, which stands at 0.864 (BCN, Library of the National Congress of Chile, 2020). The region’s employment rate reaches 57.9% (Metropolitan Labor Observatory, 2022). For its part, the region of La Araucanía has a poverty rate of 11.6% (MDS, Ministry of Social Development, 2023) and the lowest Human Development Index in the country, which is 0.770 (BCN, Library of the National Congress of Chile, 2020). The region’s employment rate reaches 48.4% (La Araucanía Labor Observatory, 2022).

Even during the health emergency caused by SARS-CoV-2, 235,357 workers in the Metropolitan region and 13,286 workers in the La Araucanía region were able to carry out their work functions from home (Metropolitan Labor Observatory, 2022; La Araucanía Labor Observatory, 2021). Additionally, 8.6% of the total number of employed workers in both the Metropolitan region and the La Araucanía region reported a decrease in their income (Metropolitan Labor Observatory, 2022; La Araucanía Labor Observatory, 2022).

The sample was purposively selected using quota sampling, whereby school authorities were approached based on the Vulnerability Reference Index by Establishment (IVE in Spanish) to obtain a sample that accurately represents the distribution of families according to the socioeconomic status of each commune, as indicated by the CASEN 2015 survey (MDS, Ministry of Social Development, 2015).

After school authorities granted authorization, parents were invited to participate in the study. Families who agreed to participate were assigned a trained interviewer who established communication by telephone and e-mail with a family member. The interviewers then emailed each parent a link containing the questionnaire. The questionnaires were administered in the Metropolitan Region between March and July 2021 and in the Araucanía region from June to December 2021.

The online questionnaire’s first page contained an informed consent form that each participant had to read, complete, and accept before proceeding. Each response was recorded on the QuestionPro platform (QuestionPro Inc) in a separate database for each couple member. After completing the instruments, the families received 10 USD for their participation.

For this study, only dyads of mothers and fathers with dependent paid work were selected, forming a sample of 232 dyads of fathers and mothers in the Metropolitan Region and 222 dyads in the Araucanía Region. The number of surveys included was needed to obtain reliable results, considering ten subjects for each item of the scales applied (Garson, 2008; Nunnally, 1967).

### 2.2 Instruments

To collect the data, a questionnaire consisted of sociodemographic questions regarding age, number of children, number of household members, working day, type of work, type of employment, region, city, and socioeconomic level, which was determined based on the total income of the household and its size (Asociación de Investigadores de Mercado, 2016). In addition, mothers and fathers responded to the following scales:

#### 2.2.1 Perceived workplace support for families scale (PWSF)

Instrument adapted by Matias et al. (2017). This scale comprises three items grouped into a single dimension, which measures the perception of support received at work regarding parental tasks (e.g., *“In my workplace, my daily work routine is flexible”*). Participants had to indicate their agreement with each statement using a 4-point Likert-type scale (1: never or rarely to 4: always). Matias et al. (2017) showed good internal consistency of this measure; the Cronbach’s alpha reported was 0.77 in fathers and 0.82 in mothers. This scale was used in its validated Spanish version in Chilean adults, where an omega = 0.90 in fathers and 0.89 in mothers was obtained (Schnettler et al., 2022).

#### 2.2.2 Work-family balance scale (WFB)

Instrument developed by Carlson et al. (2009). This scale comprises six items grouped into a single dimension, which measures the degree to which an individual meets negotiated role-related expectations in both work and family settings (e.g., “*I can manage the expectations that my supervisors and family have of me*”). Participants had to indicate their agreement with each statement using a 5-point Likert-type scale (1: strongly disagree to 5: strongly agree). Carlson et al. (2009) obtained a good internal consistency of this measure; the Cronbach’s alpha reported was 0.93. This scale was used in its validated Spanish version in Chilean adults, where an ordinal alpha = 0.89 was obtained (Riquelme-Segura et al., 2023).

#### 2.2.3 Job satisfaction scale (OJSS)

Instrument proposed by Agho et al. (1992). This scale comprises six items grouped into a single dimension, which assesses a person’s overall satisfaction with their job (e.g., *“I would not consider taking another type of job”*). Participants were asked to indicate their degree of agreement with each statement, using a 5-point Likert scale (1: strongly disagree to 5: strongly agree). Agho et al. (1992) obtained a good internal consistency of this measure, the Cronbach’s alpha reported was 0.90. This scale was used in its Spanish version validated in Chilean adults, where an omega = 0.91 in fathers and 0.91 in mothers was obtained (Schnettler et al., 2020).

#### 2.2.4 Satisfaction with family life scale (SWFaL)

Instrument proposed by Zabriskie and McCormick (2003), which corresponds to an adaptation of the Satisfaction with Life Scale (SWLS, Diener et al., 1985), in which the word “life” is replaced by “family life” in all items. This scale consists of five items grouped into a single dimension, which assesses cognitive judgments about one’s family life (e.g., “*In many respects, my family life is close to ideal*”). Participants had to indicate their degree of agreement with each statement using a 6-point Likert scale (1: completely disagree to 6: completely agree). Zabriskie and McCormick (2003) obtained a good internal consistency of this measure; the Cronbach’s alpha reported was 0.93. This scale was used in its validated Spanish version (Schnettler et al., 2017) in Chilean adults. Schnettler et al. (2020) reported an omega = 0.92 in fathers and 0.94 in mothers.

### 2.3 Data analysis

The descriptive analysis of the sample was performed using the SPSS 23.0 statistics program to characterize the study participants. Analyses of the relationships between variables were performed using the Mplus 7.11 program, using structural equation modeling (SEM) to determine the relationships between the independent and dependent variables. A robust weighted least square mean and variance adjusted (WLSMV) was chosen for variables with ordinal response.

A reliability analysis, such as the internal consistency of the instruments, was carried out by calculating the omega coefficient (McDonald, 1999). The convergent validity of the instruments was evaluated based on the standardized factor loadings of each scale (> 0.50), as well as their significance and average variance extracted (AVE > 0.50) (Hair et al., 2006). Discriminant validity was obtained by comparing the AVE of each scale with the square of the correlation between the scales (Hair et al., 2006).

The dyadic analysis was performed through an Actor-Partner Interdependence Model (Kashy and Kenny, 1999; Kenny, 1996; Kenny et al., 2006) using an Actor-Partner Interdependence Mediation Model (APIMeM) (Ledermann et al., 2011) through SEM (Cook and Kenny, 2005). The APIMeM makes it possible to determine the effects of independent variables of distinguishable dyads (actor-partner) on their dependent variables, identifying the effect of each member of the dyad on the other member, as well as assessing mediation in dyadic data by estimating the effects of the actor and the partner.

The APIM controls for different sources of non-independence, each parent’s perception of workplace family support common variance is controlled by correlating these variables for each dyad member. Correlations between the residual errors of the dependent variables and mediating variables for each family member, namely between the job and family satisfaction and work-family balance of both the father and mother, further enable the control of additional sources of actor-partner dependency (Kenny et al., 2006).

The variables region of residence, family socioeconomic status (SES), number of people in the household family members, working hours, and work modality were incorporated in the analysis with direct effects on the dependent and mediator variables, job satisfaction, family satisfaction and work-family balance, respectively, to control for their effect when estimating the fit of the data.

The fit of the model was evaluated using the comparative fit index (CFI > 0.95), the Tucker Lewis index (TLI > 0.95), and the root mean square error of approximation (RMSEA < 0.06), as proposed by Kenny et al. (2006) for dyadic analysis with SEM.

To test the mediations, the confidence intervals below and above 2.5% of the estimation were considered not to include zero, indicating a significant indirect effect (MacKinnon et al., 2007).

## 3 Results

### 3.1 Descriptive

Table 1 also shows the mean scores of perceived workplace support for families (PWSF), work-family balance (WFB), job satisfaction (OJSS), and satisfaction with family life (SWFaL).

Regarding differences between regions, couples in the Araucanía region had fewer members (*p* < 0.001) and children (*p* < 0.05) per family than couples in the Metropolitan region. The Araucanía region had a greater proportion of families belonging to the low SES, whereas the Metropolitan region had more families belonging to the high SES (*p* < 0.001). Finally, in the Araucanía region, a greater proportion of mothers worked in person, while in the Metropolitan region, a more significant proportion worked from home (*p* < 0.001). The same was true for fathers (*p* < 0.001). Regarding the variables PWSF, WFB, OJSS, and SWFaL of fathers and mothers, no statistically significant differences were identified between regions.

Table 2 presents the mean scores and correlations of the variables examined in this study. In addition, Table 2 presents the reliability of each scale using the omega coefficient; in all cases, values were above 0.70 for both mothers and fathers, indicating good internal consistency of the instruments. Convergent validity was supported by the size of the factor loadings, which were statistically significant and had values greater than 0.50 for both mothers and fathers. Discriminant validity was demonstrated by obtaining AVE values greater than the quadratic correlation between the scales in mothers and fathers.

**Table 2:** Descriptive statistics and correlations observed in different-gender dual-earner couples for the variables workplace family support perception (PWSF), work-family balance (WFB), job satisfaction (OJSS) and satisfaction with family life (SWFaL).

Scale	Factor loading range	OMEGA	AVE	1	2	3	4	5	6	7	8
1. Mothers’PWSF	0.856–0.910	0.911	0.775	–	0.067	0.050	0.006	0.127	0.010	0.008	0.007
2. Fathers’ PWSF	0.882–0.874	0.900	0.751	0.260***	–	0.023	0.110	0.027	0.119	0.022	0.036
3. Mothers’WFB	0.750–0.864	0.920	0.660	0.224***	0.153**	–	0.119	0.106	0.022	0.076	0.043
4. Fathers’WFB	0.724–0.914	0.939	0.723	0.080	0.332***	0.346***	–	0.024	0.097	0.082	0.101
5. Mothers’OJSS	0.624–0.927	0.925	0.676	0.357***	0.165***	0.326***	0.156**	–	0.073	0.022	0.010
6. Fathers’OJSS	0.623–0.928	0.934	0.707	0.100*	0.345***	0.151**	0.312***	0.271**	–	0.017	0.046
7. Mothers’ SWFaL	0.744–0.915	0.934	0.740	0.094*	0.150**	0.276***	0.288***	0.150**	0.132**	–	0.112
8. Fathers’ SWFaL	0.851–0.929	0.951	0.795	0.089	0.192***	0.209***	0.319***	0.100*	0.216***	0.335***	–

Lower part correlations/Upper part correlations squared. *p < 0.05, **p < 0.01.

### 3.2 APIM results: testing of the actor-partner hypothesis

**Figure 2:**
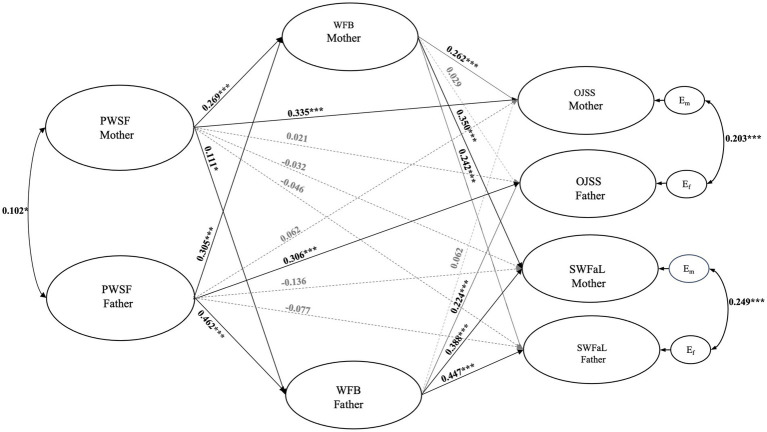
Actor-partner interdependence model of the effect of workplace family support perception (PWSF), work-family balance (WFB), job satisfaction (OJSS), and satisfaction with family life (SWFaL) in different-gender dual-earner parents. *p < 0.05, **p < 0.01, ***p < 0.001. For simplicity of the model, control variables are not reported. RMSEA = 0.044; CFI = 0.978; TLI = 0.975.

The results of the structural model estimation are shown in Figure 2. The model that assessed the association between mothers’ and fathers’ PWSF and their levels of WFB, OJSS, and SWFaL showed a good fit with the data (CFI = 0.978; TLI = 0.975; RMSEA = 0.044). A significant correlation (covariance) was found between the PWSF of both dyad members (*r* = 0.102, *p* = 0.048) as well as between the residual errors of mothers’ and fathers’ OJSS (*r* = 0.203, *p* < 0.001) and SWFaL (*r* = 0.249, *p* < 0.001).

As for control variables, mothers’ WFB was higher in families with a higher SES (*γ* = 0.185, *p* = 0.002). The WFB score was lower for fathers working 45 h per week (*γ* = −0.104, *p* = 0.046). The WFB was lower for mothers who worked in person (γ = −0.126, *p* = 0.026). Mothers’ OJSS was lower for those who worked in person (*γ* = −0.110, *p* = 0.038). Finally, mothers’ SWFaL was higher when fathers worked remotely (*γ* = 0.149, *p* = 0.003). No other significant paths between control variables and mediator or dependent variables were found (Table 3).

**Table 3:** Standardized effect estimates of control variables on work-family balance (WFB), job satisfaction (OJSS), and satisfaction with family life (SWFaL) in a sample of different-gender dual-earner parents.

Control variable → Dependent variable	Estimate	*P-value*
City→ Mothers’ WFB	0.053	0.363
City→ Fathers’ WFB	0.078	0.190
City→ Mothers’ OJSS	−0.028	0.613
City→ Fathers’ OJSS	−0.052	0.337
City→ Mothers’ SWFaL	−0.008	0.873
City→ Fathers’ SWFaL	0.080	0.143
Family socioeconomic status→ Mothers’ WFB	0.185	0.002**
Family socioeconomic status→ Fathers’ WFB	0.107	0.060
Family socioeconomic status→ Mothers’ OJSS	0.069	0.184
Family socioeconomic status→ Fathers’ OJSS	0.079	0.137
Family socioeconomic status→ Mothers’ SWFaL	0.085	0.121
Family socioeconomic status→ Fathers’ SWFaL	0.020	0.717
Number of family members→ Mothers’ WFB	−0.032	0.557
Number of family members→ Fathers’ WFB	0.003	0.952
Number of family members→ Mothers’ OJSS	0.062	0.179
Number of family members→ Fathers’ OJSS	0.080	0.090
Number of family members→ Mothers’ SWFaL	0.047	0.328
Number of family members→ Fathers’ SWFaL	−0.020	0.677
Mothers’ working hours→ Mothers’ WFB	0.075	0.167
Mothers’ working hours→ Fathers’ WFB	0.078	0.141
Mothers’ working hours→ Mothers’ OJSS	−0.030	0.532
Mothers’ working hours→ Fathers’ OJSS	0.043	0.381
Mothers’ working hours→ Mothers’ SWFaL	0.034	0.473
Mothers’ working hours→ Fathers’ SWFaL	−0.015	0.765
Fathers’ working hours→ Mothers’ WFB	−0.023	0.664
Fathers’ working hours→ Fathers’ WFB	−0.104	0.046*
Fathers’ working hours→ Mothers’ OJSS	−0.022	0.641
Fathers’ working hours→ Fathers’ OJSS	0.012	0.790
Fathers’ working hours→ Mothers’ SWFaL	0.003	0.947
Fathers’ working hours→ Fathers’ SWFaL	−0.048	0.343
Mothers’ work modality→ Mothers’ WFB	−0.126	0.026*
Mothers’ work modality→ Fathers’ WFB	−0.039	0.500
Mothers’ work modality→ Mothers’ OJSS	−0.110	0.038*
Mothers’ work modality→ Fathers’ OJSS	0.008	0.887
Mothers’ work modality→ Mothers’ SWFaL	−0.006	0.912
Mothers’ work modality→ Fathers’ SWFaL	−0.059	0.287
Fathers’ work modality→ Mothers’ WFB	−0.056	0.333
Fathers’ work modality→ Fathers’ WFB	−0.061	0.311
Fathers’ work modality→ Mothers’ OJSS	0.000	0.996
Fathers’ work modality→ Fathers’ OJSS	0.023	0.664
Fathers’ work modality→ Mothers’ SWFaL	0.149	0.003**
Fathers’ work modality→ Fathers’ SWFaL	0.079	0.141

*p < 0.05, **p < 0.01, ***p < 0.001.

As shown in Figure 2, the standardized path coefficients indicate that mothers’ (*γ* = 0.269, *p* < 0.001) and fathers’ (*γ* = 0.462, *p* < 0.001) PWSF was positively associated with their own WFB, confirming H1. Similarly, mothers’ PWSF was positively associated with fathers’ WFB (*γ* = 0.111, *p* = 0.022), and fathers’ PWSF was also positively associated with mothers’ WFB (*γ* = 0.305, *p* < 0.001), confirming H2.

On the other hand, path coefficients indicate that mothers’ (*γ* = 0.335, *p* < 0.001) and fathers’ (*γ* = 0.306, *p* < 0.001) PWSF were positively associated with their own OJSS, confirming H3. However, mothers’ PWSF was not significantly associated with fathers’ OJSS (*γ* = 0.021, *p* = 0.679), just as fathers’ PWSF was also not significantly associated with mothers’ OJSS (*γ* = 0.062, *p* = 0.315); therefore, H4 does not hold.

Similarly, mothers’ (*γ* = −0.032, *p* = 0.534) and fathers’ (*γ* = −0.077, *p* = 0.268) PWSF were not statistically associated with their SWFaL, failing to support H5. Mothers’ PWSF was not significantly associated with fathers’ SWFaL (*γ* = −0.046, *p* = 0.376), and fathers’ PWSF was not significantly associated with mothers’ SWFaL (*γ* = −0.136, *p* = 0.074), thus not supporting H6.

The path coefficients indicate that mothers’ (*γ* = 0.262, *p* < 0.001) and fathers’ (*γ* = 0.224, *p* < 0.001) WFB were positively associated with their own OJSS, confirming H7. In contrast, mothers’ WFB was not significantly associated with fathers’ OJSS (*γ* = 0.029, *p* = 0.586), and fathers’ WFB was also not positively associated with mothers’ OJSS (*γ* = 0.062, *p* = 0.227); thus, H8 is not supported.

Finally, mothers’ (*γ* = 0.350, *p* < 0.001) and fathers’ (*γ* = 0.447, *p* < 0.001) WFB was positively associated with their own SWFaL, confirming H9. Similarly, mothers’ WFB was positively associated with fathers’ SWFaL (*γ* = 0.242, *p* < 0.001), and fathers’ WFB was positively associated with mothers’ SWFaL (*γ* = 0.388, *p* < 0.001), confirming H10.

### 3.3 Indirect effects of PWSF on SWFaL and OJSS via WFB

To assess the indirect relationship between the perception of workplace family support and job and family satisfaction, the mediating role of the work-family balance was tested in both partners (actor and partner effect) (Table 4).

**Table 4:** Mediating role of work-family balance in the relationship between workplace family support perception (PWSF), job satisfaction (OJSS) and satisfaction with family life (SWFaL) for mothers and fathers.

Indirect effects	Estimate	Lower 2.5%	Upper 2.5%	*P-value*
Mothers’ PWSF→ Mothers’ WFB → Mothers’ OJSS	0.070	0.037	0.104	<0.001***
Fathers’ PWSF→ Fathers’ WFB → Fathers’ OJSS	0.103	0.055	0.151	<0.001***
Mothers’ PWSF→ Fathers’ WFB → Mothers’ OJSS	0.007	−0.006	0.019	0.279
Fathers’ PWSF→ Mothers’ WFB → Mothers’ OJSS	0.080	0.040	0.120	<0.001***
Fathers’ PWSF→ Fathers’ WFB → Mothers’ OJSS	0.028	−0.018	0.075	0.231
Mothers’ PWSF→ Mothers’ WFB → Fathers’ OJSS	0.008	−0.021	0.037	0.589
Mothers’ PWSF→ Fathers’ WFB → Fathers’ OJSS	0.025	0.001	0.049	0.042*
Fathers’ PWSF→ Mothers’ WFB → Fathers’ OJSS	0.009	−0.023	0.041	0.583
Mothers’ PWSF→ Mothers’ WFB → Mothers’ SWFaL	0.094	0.053	0.135	< 0.001***
Fathers’ PWSF→ Fathers’ WFB → Fathers’ SWFaL	0.206	0.145	0.267	< 0.001***
Fathers’ PWSF→ Mothers’ WFB → Fathers’ SWFaL	0.074	0.032	0.116	0.001**
Mothers’ PWSF→ Fathers’ WFB → Mothers’ SWFaL	0.043	0.003	0.083	0.033*
Fathers’ PWSF→ Mothers’ WFB → Mothers’ SWFaL	0.107	0.055	0.159	< 0.001***
Fathers’ PWSF→ Fathers’ WFB → Mothers’ SWFaL	0.179	0.112	0.246	< 0.001***
Mothers’ PWSF→ Mothers’ WFB → Fathers’ SWFaL	0.065	0.029	0.101	< 0.001***
Mothers’ PWSF→ Fathers’ WFB → Fathers’ SWFaL	0.050	0.006	0.094	0.026**

PWSF: Workplace family support perception. WFB: Work-family balance. OJSS: Job satisfaction. SWFaL: Satisfaction with family life. *p < 0.05, **p < 0.01, ***p < 0.001.

Regarding actor effects, the mediating role of mothers’ WFB in the relationship between their own PWSF and OJSS was verified because the confidence interval excluded zero, indicating a significant indirect effect (standardized indirect effect = 0.070, 95% CI = 0.037, 0.104). The mediating role of fathers’ WFB between their PWSF and OJSS was also supported (standardized indirect effect = 0.103, 95% CI = 0.055, 0.151).

Regarding partner effects, mothers’ WFB mediated the relationship between fathers’ PWSF and mothers’ OJSS (standardized indirect effect = 0.080; 95% CI = 0.040; 0.120). The mediating role of fathers’ WFB between mothers’ PWSF and fathers’ OJSS was also supported (standardized indirect effect = 0.025, 95% CI = 0.001, 0.049). No other significant indirect effects of WLB between workplace family support perception and job satisfaction were found; therefore, hypothesis 11 is partially satisfied.

In contrast, the mediating role of the WFB between PWSF and SWFaL in actor and partner effects was fulfilled in all possible options. Regarding actor effects, the mediating role of mothers’ WFB in the relationship between their own PWSF and SWFaL was confirmed (standardized indirect effect = 0.094, 95% CI = 0.053, 0.135). The mediating role of fathers’ WFB between their PWSF and SWFaL was also verified (standardized indirect effect = 0.206, 95% CI = 0.145, 0.267).

As for partner effects, the mediating role of mothers’ WFB on the relationship between fathers’ PWSF and fathers’ SWFaL was confirmed (standardized indirect effect = 0.074; 95% CI = 0.032; 0.116). The mediating role of fathers’ WFB on the relationship between mothers’ PWSF and SWFaL was also significant (standardized indirect effect = 0.043, 95% CI = 0.003, 0.083). The mediating role of mothers’ WFB between fathers’ PWSF and mothers’ SWFaL was confirmed (standardized indirect effect = 0.107; 95% CI = 0.055; 0.159). Likewise, the mediating role of fathers’ WFB in the relationship between fathers’ PWSF and mothers’ SWFaL was confirmed (standardized indirect effect = 0.179; 95% CI = 0.112; 0.246). The mediating role of mothers’ WFB between mothers’ PWSF and fathers’ SWFaL was also supported (standardized indirect effect = 0.065, 95% CI = 0.029, 0.101). Similarly, the mediating role of fathers’ WFB in the relationship between mothers’ PWSF and fathers’ SWFaL was supported (standardized indirect effect = 0.050, 95% CI = 0.006, 0.094). Hence, hypothesis 12 is confirmed.

## 4 Discussion

This study shows the direct and indirect links between perceptions of workplace family support and work satisfaction, as well as the indirect link through family satisfaction. Work-family balance was therefore proven to be a very important mediator in this process. Another set of innovative findings shows the crossover effects of workplace family support between parents, enhancing work-family balance, and the crossover effect of work-family balance on family support. The findings obtained are discussed below.

### 4.1 Workplace family support perception, work-family balance, job satisfaction, and family satisfaction

Our results showed that the perception of workplace family support is positively and significantly associated with the work-family balance in mothers and fathers in the actor and the partner, thus confirming our H1 and H2.

The perception of workplace family support is a work resource (Matias et al., 2017) that aids mothers and fathers in navigating the work-family interface (Yucel and Minnotte, 2017), enabling them to achieve a more effective balance between their professional and domestic responsibilities (Lo Presti et al., 2022). Therefore, contextual resources generated at work allow them to cope with the demands of work and family by acquiring the conditions needed to manage time or do their job more efficiently, accumulating resources in the work environment that could be invested directly in the family environment.

The accumulation of resources resulting from the positive relationship in mothers and fathers between workplace support for the family and the work-family balance makes it possible to create resource caravans (Hobfoll, 1989, 2011) and thus improve the balance between work and family roles. This indicates that the labor resources obtained are sufficient to adequately manage work and family roles in ecological conditions that promote resource sustainability (Hobfoll et al., 2018). Similarly, accumulating resources were found to enable symmetrical interindividual resource transfer, as suggested by the Crossover Model of Chen et al. (2015). This finding contributes to filling a gap in the existing literature by demonstrating that the resources acquired in the workplace by mothers’ transfer to their partners’ ability to manage and balance their work and family roles, and the same holds for fathers.

The transfer of resources is based on interdependence, as proposed by Murray Bowen’s Family Systems Theory (1978), due to the emotional relationship between mothers and fathers, which causes a reciprocal influence on each other’s thoughts, emotions, and behaviors (Kerr and Bowen, 1988). Consequently, the support that a mother or father receives enhances both their and their partner’s wellbeing, as it facilitates the allocation of sufficient resources to various roles and activities, consequently promoting balanced management of work and family obligations.

The results can further be interpreted within the Chilean labor context, which offers optional measures to assist families, including extraordinary leaves for family matters, hour banks, and other initiatives. These measures are subject to voluntary negotiation between organizations and their employees (Gómez-Urrutia and Jiménez-Figueroa, 2019) but can improve the work-family balance of the other member of the couple.

Our results also confirmed that the perception of workplace family support is directly and positively associated with job satisfaction in both mothers and fathers, confirming H3. However, this each workplace resource was not significantly directly associated with the other partner’s job satisfaction neither in mothers nor fathers, thus not confirming H4.

Resources generated at work increase job satisfaction (Mascarenhas et al., 2022) because obtaining contextual resources such as workplace support for the family enables the activation of a process of gain and accumulation of resources (Hobfoll, 1989) to cope with work demands and achieve greater job satisfaction. Workplace support for the family is more strongly linked to work outcomes since it can elicit good emotional responses and behavioral outcomes that enhance job satisfaction (Zhang et al., 2018).

However, the support resources obtained at work by fathers and mothers are not transferred to increase the job satisfaction of the other member’ of the couple. A possible explanation for this result may be the weak correlation between fathers’ and mothers’ perceptions of workplace family support from different employment sources. Therefore, it is less likely that one partner’s job support would increase the other’s job satisfaction.

Regarding the expected direct relationship between perceptions of workplace family support and family satisfaction, our results do not confirm this effect (H5 actor effect, H6 partner effect). Although support provided by organizations has been shown to increase family efficacy and functioning (Gahlawat et al., 2019; Mesimo-Ogunsanya, 2017; Tang et al., 2014), our results indicate that it does not directly increase family satisfaction. Similarly, the results contradict the evidence of Brady et al. (2021), who suggest that resources derived from workplace family support perception can be transferred directly between partners, enhancing their family experience.

These results could be viewed as a consequence of current Chilean public policies because they have been predominantly geared toward protecting maternity and care in the work setting (Gómez-Urrutia and Jiménez-Figueroa, 2019). Consequently, a wider range of favorable work resources would not be available to stimulate the profit spiral and resource accumulation process (Hobfoll, 1989), thereby impacting the potential for directly increasing the valuation of their own family life and that of their partner.

However, the results indicate that work-family balance was positively and significantly associated with job satisfaction in mothers and fathers (H7 actor effect). In contrast, the work-family balance of one partner was not significantly associated with the job satisfaction of the other in both mothers and fathers (H8 partner effect).

The findings regarding the direct relationship between work-family balance and job satisfaction among mothers and fathers align with previous research conducted in Chile (Riquelme-Segura et al., 2023; Chiang Vega et al., 2024) as well as studies from other countries (Zhang et al., 2020; Leung et al., 2020). Compliance with negotiated expectations related to professional and family roles (Grzywacz and Carlson, 2007) enhances work performance, which in turn influences the assessment of one’s work experience. According to the COR Theory (Hobfoll, 1989), mothers and fathers who manage various work and family roles help allocate resources to the work setting, thereby increasing their job satisfaction.

The non-significant association between one partner’s work-family balance and the other partner’s job satisfaction reinforces the notion that job satisfaction may depend more on factors related to the job itself (Agho et al., 1992) than on the work-family balance experienced by the partner.

Finally, the results show that the work-family balance is positively and significantly associated with family satisfaction in mothers and fathers (H9 actor effect, H10 partner effect). These results are consistent with previous international research (Carlson et al., 2009; Ferguson et al., 2012; Leung et al., 2020), particularly in Chilean workers at the individual level (Riquelme-Segura et al., 2023). Although the international literature reports crossover effects of work-family balance between US workers and their spouses (Wan et al., 2021), our results contribute to the knowledge generated in the Chilean population by demonstrating the interindividual transfer or crossover symmetrical of resources between members of a dyad in the relationship between work-family balance and family satisfaction.

The achievement of role-related expectations negotiated and shared between an individual and their role partner in the work and family environment (Grzywacz and Carlson, 2007) is supported by Marks and MacDermid’s (1996) role balance theory, which proposes that role balance is not an outcome, but rather it can generate resources to be invested in the family and thus increase family satisfaction. These approaches make it possible to understand the direct relationship between work-family balance and family satisfaction at the individual level, as well as the crossover to the partners.

Furthermore, it is essential to acknowledge that the data were collected during a health crisis, wherein individuals focused on maintaining order and safeguarding family life due to circumstances such as school closures (Macartney et al., 2020). Consequently, parents may exhibit an unequal commitment to their responsibilities while balancing professional obligations and family life, enabling them to value family life positively. Flexible work measures could contribute to understanding these relationships, as they are labor resources that can be managed to achieve greater subjective wellbeing at the family level.

### 4.2 Work-family balance as a mediator

No previous studies were identified in the literature review that evaluates the mediating role of work-family balance in the relationship between workplace family support perception and job satisfaction. In this regard, using the APIMeM model, our results demonstrated the mediating role of the work-family balance, in the actor and partially in the partner, in the relationship between workplace family support perception and job satisfaction in mothers and fathers (H11).

As expected, the work-family balance acts as an underlying mechanism to explain the relationship between the perception of workplace family support and job satisfaction in the actor and partially in the partner. This means that the perception of workplace family support is directly linked to job satisfaction and the work-family balance in mothers and fathers. This reveals the importance of supportive work environments, as they are particularly beneficial for dual-income families (Lawson et al., 2019) by allowing them to increase their available resources and thus achieve a better work-life balance. In this way, consistent with COR Theory (Hobfoll, 1989), mothers and fathers could improve their levels of job satisfaction.

The partner mediates the work-family balance, influenced by asymmetrical relationships and potentially perpetuated by persistent gender gaps (Ministry of Women and Gender Equity, 2021). The results indicate that the perception of support for the family in the father’s workplace impacts the mother’s work-family balance and then the mother’s job satisfaction. Similarly, the perception of workplace family support in the mother’s workplace first affects the father’s work-family balance and then the father’s job satisfaction.

Increased job support from one partner allows the other partner to accumulate resources to improve their job and family performance and increase job satisfaction. These findings underscore the importance of offering support to employed mothers and fathers, as the resources generated from employment positively influence their partners through a within-domain effect (Amstad et al., 2011), thereby enhancing work-family balance and the partner’s job satisfaction.

However, contrary to expectations, the workplace family support perception and the mother’s job satisfaction were not indirectly related through the father’s work-family balance. Similarly, the workplace family support perception and the father’s job satisfaction are not indirectly related through the mother’s work-family balance. The results indicate that work-family balance is not an interindividual mediating variable in the relationship between one partner’s workplace family support perception and the other partner’s job satisfaction. This could be due to the different sources of work that generate the labor resource and the low relationship between the two workplace family support perceptions. The work-family balance plays a limited role in this relationship since job satisfaction is attributed more to a person’s appraisal of their own work (Agho et al., 1992) than to the work-family balance experienced by their partner.

There is a paucity of research on the mediating effect of work-family balance on the relationship between workplace family support perception and family satisfaction (Lo Presti et al., 2020). The results demonstrate the mediating role of the work-family balance, in the actor and the partner, in the relationship between the workplace family support perception and family satisfaction in mothers and fathers (H12). A cross-domain effect (Frone et al., 1992), at the intra- and interindividual levels, is recognized in the relationship between workplace family support perception and family satisfaction through the work-family balance.

Therefore, the work-family balance is an underlying mechanism explaining the relationship between the perception of workplace family support and family satisfaction in the actor and the partner. Therefore, although in this study, the workplace family support perception is not directly linked to family satisfaction in fathers and mothers, it is connected to the ability to negotiate, do a good job, achieve expectations, and fulfill work and family responsibilities at the intra- and interindividual levels.

The results obtained are consistent with the findings of Lo Presti et al. (2020) in Italian dual-earner couples on the existence of a mediating role of work-family balance between work-family organizational support and family satisfaction and of cross-effects of work-family balance on the couple’s family satisfaction. In addition, it helps to identify the existence of cross-domain effects (Frone et al., 1992), so it is suggested that the work-family balance should not be reduced to a family problem that affects only the individual level.

The work-family balance allows both mothers and fathers in dual-income families to increase their appreciation of their work experiences and perform better in the family setting. Consequently, innovative organizational structures in the workforce that align with family dynamics are needed, enabling both mothers and fathers to fulfill their professional and family obligations without having to prioritize one over the other. This requires a commitment from institutions or companies to support family wellbeing, as this could foster a mutually beneficial and positive relationship.

## 5 Limitations

This study has limitations and should be considered in future research. Despite our conceptual model being fully informed by robust theoretical models, the cross-sectional design does not permit the establishment of causal relationships. Therefore, these relationships should be evaluated further in future longitudinal studies. Likewise, the study data were obtained through self-reporting, so the responses given in the instrument could have been influenced by social desirability. This generates a self-report bias, where participants may report higher scores than those they actually experience in terms of work support, work-family balance, job satisfaction, or family satisfaction. Although we recruited families using quota sampling, the sample is non-probabilistic and consists of parents from only two regions of a single country, thereby limiting the generalizability of these results. Consequently, their findings address a specific cultural context. Lastly, although the region of residence was included as a control variable, the application of the survey in different months in Temuco and Santiago may have influenced the responses, considering the distinct circumstances in different periods of time.

Despite the aforementioned limitations, this study makes a valuable contribution to the existing literature on the positive direct and indirect associations between workplace family support perception, work-family balance, job satisfaction, and family satisfaction.

### 5.1 Implications and future studies

Theoretically, the study provides evidence that the work-family balance acts as a mediator within relationships and to a greater extent in cross domain relationships, in addition to those proposed by Amstad et al. (2011) and Frone et al. (1992) that allude to enrichment and work–family conflict.

From a practical perspective, these study results contribute to exploring family wellbeing by suggesting that the resources earned or accumulated by an individual at work can enhance work-family balance, benefiting both the individual and their partner. Having scarce or insufficient resources, such as a lack of flexible hours, limited leave for medical or school appointments, or other work-related difficulties, causes both mothers and fathers to struggle with balancing the demands of work and family, ultimately affecting their wellbeing. This knowledge can be applied in new crisis or health emergency contexts to facilitate the prompt implementation of labor support practices that enhance the wellbeing of workers.

Consequently, it is imperative for labor-related state agencies, including companies and institutions, to implement labor practices that facilitate the activation of resource acquisition and accumulation among their employees, thereby promoting work-family balance, which would enhance both job satisfaction and family satisfaction at the individual and organizational levels. This study emphasizes the importance of family-friendly labor policies, particularly in the context of pandemics or health emergencies, that incorporate a gender perspective, offering support for both men and women to achieve a balance in their roles.

Future research should investigate alternative forms of workplace support for families, such as supervisor or coworker support, as a resource that facilitates the accumulation and gain of resources at both intra- and interpersonal levels, as described by Hobfoll (1989). It is essential to investigate potential moderators that may influence the relationship between organizational support for families, work-family balance, and satisfaction in both work and family life among employed mothers and fathers. This examination is particularly relevant in light of the changes in work and family dynamics that have occurred since the COVID-19 pandemic, including factors such as socioeconomic status.

## 6 Conclusion

In recent decades, research on organizational support and its positive impact on individuals, which reduces psychological pressure and promotes a balance between work and family domains, has expanded (Drummond et al., 2017; Jimenez-Figueroa et al., 2024; Pattusamy and Jacob, 2017; Yucel and Minnotte, 2017). Exploring the management of instrumental and affective resources derived from the work domain is crucial for understanding how the accumulation of resources, as proposed by Hobfoll (1989), facilitates the achievement of work-family balance and enhances individual wellbeing in both work and family settings. However, it is also relevant to study the crossover or transfer of resources to partners due to the interdependence between individuals who share the same environment, as outlined in the tenets of Bowen’s Family Systems Theory (1978).

In this regard, the study provides new insights into the direct and indirect positive relationships between workplace family support perceptions, work-family balance, and both job and family satisfaction, highlighting the symmetrical crossover effects in mothers and fathers. They specifically show how work-family balance serves as a key mechanism for achieving higher job satisfaction and family satisfaction among employed mothers and fathers.

The text discusses how it identifies matching effects within domains (Amstad et al., 2011) and across different domains (Frone et al., 1992). It highlights that cross-domain resource transfers play a significant role in mediating the relationship between perceptions of workplace family support and family satisfaction.

Finally, although this research provides knowledge in a COVID19 context, this knowledge is relevant for future health emergencies, since learning from past crises allows us to better face other crises (Mutch, 2020; Pascual et al., 2024).

## Data Availability

The data analyzed in this study is subject to the following licenses/restrictions: will be provided upon request. Requests to access these datasets should be directed to leonor.riquelme@ufrontera.cl.
